# Bioacoustic Detection of Wolves Using AI (BirdNET, Cry-Wolf and BioLingual)

**DOI:** 10.3390/ani16020175

**Published:** 2026-01-07

**Authors:** Johanne Holm Jacobsen, Pietro Orlando, Line Østergaard Jensen, Sussie Pagh, Cino Pertoldi

**Affiliations:** 1 Department of Chemistry and Bioscience, Aalborg University, 9220 Aalborg, Denmark; line.oestergaard.jensen@gmail.com (L.Ø.J.); sup@bio.aau.dk (S.P.); cp@bio.aau.dk (C.P.); 2 Department of Agriculture, Mediterranean University of Reggio Calabria, 89124 Reggio Calabria, Italy; rlnptr00l20f112a@studenti.unirc.it; 3 Aalborg Zoo, Mølleparkvej 63, 9000 Aalborg, Denmark

**Keywords:** acoustic monitoring, bioacoustics, *Canis lupus*, wolf howls

## Abstract

Rising numbers of wolf (*Canis lupus*) populations make traditional, resource-intensive methods of wolf monitoring increasingly challenging and often insufficient. This study explores how wolf howls can be used as a new monitoring tool for wolves by applying Artificial Intelligence (AI) methods to detect and classify wolf howls from acoustic recordings, thereby improving the effectiveness of wolf population monitoring. Three AI approaches are evaluated: BirdNET, Yellowstone’s Cry-Wolf project system, and BioLingual. Data were collected using Song Meter SM4 (SM4) audio recorders in a known wolf territory in Klelund Dyrehave, Denmark, and manually validated to establish a ground truth of 260 wolf howls. Results demonstrate that while AI solutions currently do not achieve the complete precision or overall accuracy of expert manual analysis, they offer tremendous efficiency gains, significantly reducing processing time. BirdNET achieved the highest recall at 78.5% (204/260 howls detected), though with a low precision of 0.007 (resulting in 28,773 false positives). BioLingual detected 61.5% of howls (160/260) with 0.005 precision (30,163 false positives), and Cry-Wolf detected 59.6% of howls (155/260) with 0.005 precision (30,099 false positives). Crucially, a combined approach utilizing all three models achieved a 96.2% recall (250/260 howls detected). This suggests that while AI solutions primarily function as powerful human-aided data reduction tools rather than fully autonomous detectors, they represent a valuable, scalable, and non-invasive complement to traditional methods in wolf research and conservation, making large-scale monitoring more feasible.

## Simple Summary

Assessment of wolf (*Canis lupus*) populations today relies on multiple time-consuming and resource-intensive methods, including DNA testing of feces and wolf kills and wolf observations on wildlife cameras. This study aimed to explore wolf howls as an alternative monitoring tool for wolves and to compare several Artificial Intelligence (AI) methods against a baseline of manual registration for detecting and classifying wolf howls from audio recordings. The results show that AI-based methods like BirdNET, BioLingual, and Cry-Wolf achieved high detection rates (78.5%, 61.5%, and 59.6% recall—the proportion of actual howls successfully detected—respectively), though they also produced a substantial number of false positives. Crucially, combining these AI methods yielded an impressive 96.2% recall for actual howls. The use of automated AI methods significantly reduced the time spent on analysis of recordings, enabling the processing of larger datasets with fewer resources. This study demonstrates how the integration of AI-driven acoustic analysis can act as a non-invasive and efficient method, holding the possibility of becoming a standard for monitoring wolf populations and many other animal species.

## 1. Introduction

The wolf population in Denmark has grown from the first confirmed wolf in 2012 to approximately 50 individuals by 2025, representing a steady recolonization across multiple territories [1,2]. This recovery has created both conservation opportunities for ecosystem restoration and practical management challenges for Danish wildlife authorities [3,4].

Denmark’s wolf monitoring has historically relied on a collaborative framework between the Danish Environmental Protection Agency (Agency for Green Land Conversion and Aquatic Environment) and Aarhus University. The traditional approach combines DNA analysis from scat samples and saliva collected from wounds of animal killed by wolves, wildlife camera trapping, and citizen-reported sightings [5,6]. However, these intensive monitoring protocols were discontinued in 2025 due to escalating costs as wolf numbers increased, with DNA analysis now limited to approximately 100 samples annually [7].

The current monitoring strategy has shifted toward pack-based population estimates using a conversion factor of 7 individuals per confirmed pack, introducing uncertainty due to natural variation in pack sizes and the challenge of accurately identifying all active packs across Denmark’s expanding wolf territories [7]. Given these limitations in traditional monitoring approaches, alternative methods are increasingly necessary. Acoustic monitoring presents a promising complement for wolf population assessment, offering advantages in being non-invasive, relatively inexpensive, and capable of covering large geographical areas efficiently [6,8].

Wolf howls can transmit over distances of up to 6–11 km under optimal conditions [9] and contain valuable information about pack composition [10], territorial boundaries [11], and even individual identity [12,13]. Previous research in Denmark has demonstrated the feasibility of individual wolf identification through acoustic analysis. Larsen et al. (2022) [14] successfully used multivariate analysis to distinguish between individual wolves and even different wolf subspecies from howl recordings, achieving high classification accuracy for solo howls. This groundwork established that wolf vocalizations contain sufficient individual-specific characteristics for identification purposes, suggesting that acoustic monitoring could potentially contribute to individual-level population assessment—a critical component for accurate wolf management in Denmark.

However, the manual analysis of acoustic recordings remains laborious [14], creating a bottleneck in data processing that limits broader implementation. This efficiency bottleneck has prompted interest in automated analysis solutions.

Acoustic monitoring has been successful for detection of other wildlife species, particularly in birds and bats [15,16]. These established applications demonstrate the maturity and reliability of Artificial Intelligence (AI)-driven bioacoustics analysis, providing a strong foundation for adaptation to wolf monitoring applications.

Recent advances in AI technologies offer potential solutions to the acoustic analysis challenge [17]. AI-based methods for automated detection and classification of animal vocalizations have shown promising results across various species [18], though their application to wolf monitoring, particularly in European contexts, remains an actively developing area [14,19]. The integration of these technologies could significantly enhance monitoring efficiency while maintaining necessary accuracy for conservation management decisions [20].

This study aims to compare three AI-based methods (BirdNET, Cry-Wolf, and BioLingual) against traditional manual analysis to explore their effectiveness in wolf monitoring scenarios within Danish territories. Detection accuracy, processing efficiency, resource requirements, and potential applications in conservation management are compared and evaluated. By comparing established approaches with AI-driven acoustic monitoring, the study seeks to explore how integrating AI methods can serve as a cost-effective complement to Denmark’s evolving wolf monitoring strategy.

## 2. Materials and Methods

### 2.1. Introduction to Study Species

The target species for this research is the Eurasian wolf (*Canis lupus lupus*), a subspecies representing the Central European population to which Denmark’s resident wolves belong. Since the return of wolves to Denmark in 2012, several territories have been established, with the species showing gradual population recovery across the country [7].

### 2.2. Study Area

Data collections took place between August 2021 and February 2022 at within the 1400-hectare Klelund Dyrehave, a fenced nature reserve in Southern Jutland in Denmark. The nature reserve is a publicly accessible wildlife park situated in a mixed habitat consisting of plantation forests, natural woodland, and heathland areas. This territory has been continuously occupied by wolves since August 2020, starting with a breeding pair comprising the Danish-born male and German-born female. The pair has demonstrated successful reproductive behavior, with documented litters including a minimum of four pups born in 2021 and six pups in 2022 [7]. Acoustic recordings were conducted using passive recording equipment deployed throughout the territory to capture natural vocal behaviors. From 21 August 2021 to 20 February 2022, two autonomous recorders operated for a total of 826.5 h.

### 2.3. Acoustic Data Collection

Song Meter SM4 acoustic recorders (Wildlife Acoustics Inc., Maynard, MA, USA) were used for recordings. The acoustic recorders have a sampling rate of 44.1 kHz and an amplitude resolution of 16 bits. Two recorders were placed 1.22 km apart. One recorder was positioned on a tree and one on a fence within the territory, approximately 1.5–2 m above ground. The recorders were set to auto-record continuously from dusk till dawn (17:00–07:30) as this corresponds to the period when wolves are most vocally active, and recordings were saved to SD cards with 128 GB storage capacity. All audio files were saved in WAV format to preserve recording quality for subsequent acoustic analysis.

### 2.4. Datasets for Evaluation

For the comparison of all three AI methods, four datasets were used. The datasets were separated chronologically to correspond with the recording capacity of the SD cards and battery of the recording equipment. This division served two purposes: it accommodated the logistical constraints of data retrieval and storage, and it allowed for the comparison of model performance across distinct seasonal periods. These datasets were meticulously manually annotated for wolf howls, serving as the “ground truth” for the analysis.

Across 826.5 h of audio, we confirmed 260 wolf howls:

- dataset 1 (26 October–7 November 2021), 188.5 h and 102 howls
- dataset 2 (22 November–5 December 2021), 203 h and 50 howls
- dataset 3 (7–20 February 2022), 203 h and 8 howls
- dataset 4 (28 September–13 October 2021), 232 h and 100 howls

### 2.5. Manual Annotation and Ground Truth

All acoustic data designated for evaluation underwent a rigorous process of manual annotation to establish the definitive ‘ground truth’ for wolf howl occurrences. During this process, each identified wolf’s howl was precisely marked with its start and end times. Furthermore, to provide detailed contextual information, specific labels were assigned to each howl. These annotations captured relevant environmental or acoustic conditions present during the howl, such as rain, red deer vocalizations, or unclear/faint howls. This manual annotation served as the baseline against which the performance of all automated detection methods was compared.

### 2.6. Automated Detection Methods

**Figure 1:**
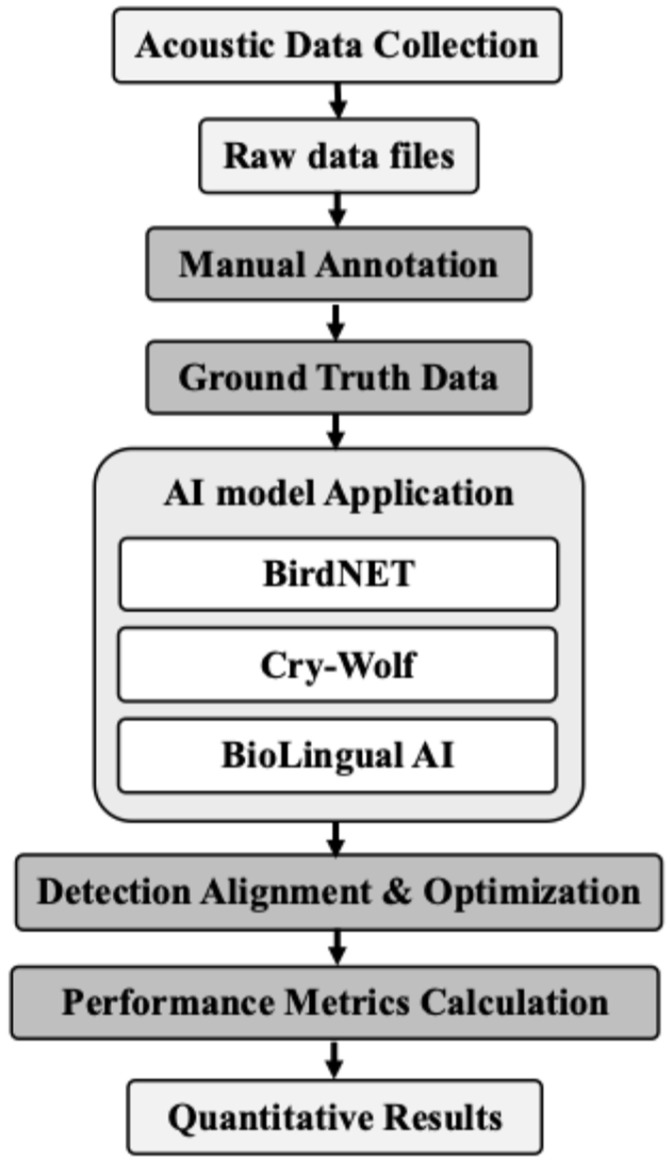
Illustrating the overall workflow of Artificial Intelligence (AI) detection and evaluation.

Figure 1 summarizes the full pipeline, tracing the process from acoustic data collection and manual annotation through model application and alignment to quantitative performance evaluation.

#### Overview of AI Systems Tested

BirdNET (Version 2.4) was originally developed as a deep learning-based system for real-time identification of avian vocalizations, utilizing convolutional neural networks trained on extensive bird song databases. While primarily designed for bird species detection, BirdNET’s extensive classification system includes over 6000 classes, among which are several mammalian species, including the wolf. The model processes audio in fixed 3 s segments.

Cry-Wolf was developed specifically for wolf vocalization detection, with initial applications in Yellowstone National Park [14]. The system operates through Kaleidoscope Pro software (Version 5.6.8) using targeted acoustic parameters: a frequency range of 200–750 Hz optimized for wolf howl fundamental frequencies, a duration range of 1.5–60 s, and FFT window size of 2.33 ms. This configuration allows the system to focus on acoustic characteristics most diagnostic of wolf howls while filtering out non-target sounds. Following acoustic feature extraction, the system employs traditional clustering methods to classify the detected segments. This approach represents a more conventional signal processing methodology compared to the deep learning-based models, relying on manually specified acoustic features rather than learned representations.

BioLingual (https://github.com/david-rx/BioLingual, accessed in 14 December 2025) represents a fundamentally different approach compared to the other models in this study, operating as a transformer-based language-audio model trained on the large-scale AnimalSpeak dataset containing over a million audio-caption pairs. Unlike traditional classification models that are trained on fixed class sets, BioLingual functions as a zero-shot classifier that can identify species calls without being explicitly trained on those specific classes. The model analyzes 10 s audio segments by finding the most similar label representation to an audio representation through cosine similarity calculations between audio embeddings and text embeddings of task label prompts. For this study, the model was provided with 350 classes derived from BirdNET’s species list for Denmark, from which it selected the most appropriate label for each audio clip. The BirdNET classes were used to ensure consistency across methods.

### 2.7. Parameter Optimization and Alignment

#### 2.7.1. Buffer Window Analysis for Detection Alignment

To account for temporal discrepancies between manual annotations and AI detections, buffer window analysis was conducted, testing 0, 3, and 10 s windows. The 10 s buffer was adopted as it significantly improved true positive capture (reducing missed detections by 23% across the three models and four datasets, while the associated increase in false positives for each model was <5%, see in Table S1).

#### 2.7.2. Confidence Threshold Selection Strategy

**Figure 2:**
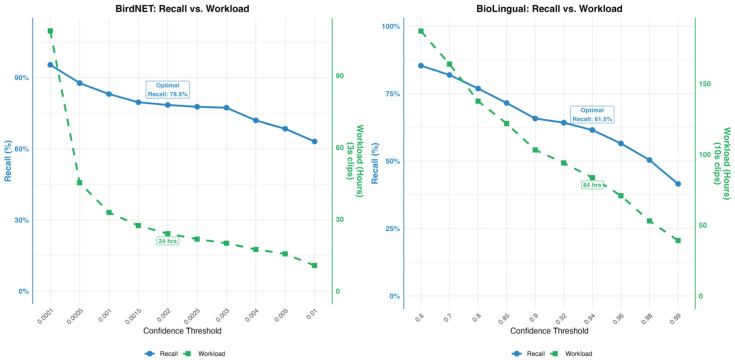
Parameter optimization curves for BirdNET and BioLingual. The plots illustrate the trade-off between Recall (Sensitivity; blue solid line) and the practical Workload in review hours (green dashed line).

To balance the competing objectives of maximizing sensitivity (Recall) and minimizing the manual verification workload (False Positives), a grid-search optimization strategy was used. A range of confidence thresholds for BirdNET (0.0001–0.01) and BioLingual (0.60–0.99) against the full annotated dataset. The performance trade-offs and the selection of the optimal thresholds are visualized in Figure 2.

#### 2.7.3. BirdNET

BirdNET’s confidence threshold was optimized through iterative testing and the optimal threshold of 0.002 was selected based on optimal recall (78.5%; 204/260 howls), while minimizing false positives (28.7%). At thresholds above 0.002 recall marginally increased but resulted in an exponential increase in workload (green dashed line). At thresholds above 0.002 the workload decreased but caused a steep decline in recall.

#### 2.7.4. BioLingual

BioLingual’s confidence threshold was optimized through iterative testing across values from 0.9 to 0.95. The optimal threshold of 0.94 was selected based on optimal recall (61.5%; 160/260 howls) while minimizing false positives (30.1%). This threshold results in a workload of 84 h. Higher thresholds caused a steep decline in recall.

#### 2.7.5. Cry-Wolf Configuration

Unlike the other two models, Cry-Wolf was run using its default pipeline with the Kaleidoscope Pro detector (200–750 Hz band-pass, 1.5–60 s event duration, 2.33 ms FFT window). To ensure comparability with the other models, we applied a uniform evaluation protocol: detections were aligned to the manual annotations using a symmetric 10 s tolerance window, and performance was scored with consistent definitions of true positives, false positives, and false negatives.

#### 2.7.6. Generalizability and Robustness

To ensure the selected thresholds were robust and not over-fitted, the optimization process was validated across four datasets representing varying acoustic conditions within Klelund Dyrehave. The recorders were placed in different habitat types; one in the forest and one in a more open location on a fence. The recordings captured variations in weather (heavy rain and wind) and seasonal differences like red deer rutting calls. These varying conditions confirms that the operational thresholds function effectively across heterogeneous soundscapes.

### 2.8. Performance Metrics

Detection performance was quantified using precision, recall, and F1-score, computed from counts of true positives, false positives, and false negatives (Table 1).

## 3. Results

### 3.1. Overall Performance on Datasets

The results demonstrate significant variability across the three AI models within the four datasets. BirdNET achieved the highest recall (78.5%). It successfully detected the greatest proportion of actual wolf howls (Table 2).

Table 3 reports model-level performance aggregated across the four datasets using a 10 s alignment window, presenting precision, recall, and F1-score; values in parentheses denote true positives over the corresponding denominator.

### 3.2. Detailed Performance by AI System and Context

#### Impact of Environmental and Acoustic Conditions

Analysis of AI performance relative to manually annotated howl characteristics reveals system-specific strengths and vulnerabilities. An analysis of the labels of each howl revealed how environmental and acoustic conditions affected detection accuracy for the methods (Table 4).

This analysis illuminates specific challenges faced by each AI system on the data, with BirdNET showing consistent high performance across all acoustic conditions (Table 5).

Across all three models, the principal limitation is very low precision (about 0.5–0.7%), yielding roughly 141–195 false positives per true positive and imposing a substantial verification workload. Cry-Wolf further shows instability—zero recall in one dataset and weak detection of faint howls—indicating sensitivity to recording conditions and temporal variation. BioLingual performs poorly in rain (25% detection) and is inconsistent even for clear howls (46.9%), with mixed outcomes under interference. BirdNET, despite high recall, is chiefly constrained by precision. Taken together, these weaknesses hinder scalability in heterogeneous soundscapes and motivate stricter post-processing, more conservative thresholding, and context-aware filtering to suppress false positives without eroding Recall.

## 4. Discussion

This study compared three AI-based methods—BirdNET, BioLingual, and Cry-Wolf—for detecting wolf howls in acoustic recordings from Dyrehave, Denmark. The recall rates observed were 78.5% for BirdNET, 61.5% for BioLingual, and 59.6% for Cry-Wolf. When combining the methods, the recall rate notably increased to 96.2%. Although all three models generated substantial numbers of false positives, they demonstrated strong potential as tools for human-assisted data reduction, especially when used together. This highlights their role not as fully autonomous detectors but as effective pre-screening solutions that can significantly streamline the manual review process, thus enhancing the feasibility of large-scale, non-invasive wolf monitoring efforts.

The following sections explore the performance characteristics and practical implications of each AI detection method in greater detail, illustrating their complementary strengths and limitations in real-world acoustic environments.

### 4.1. Performance of AI Detection Methods: Interpretation and Practical Implications

Overall, BirdNET demonstrated the highest recall (78.5% across 260 howls), identifying 204 out of 260 manually confirmed howls. This positions BirdNET as the most suitable choice for initial screening in projects where the priority is to identify most potential wolf howls. Its strength in recall is evident across various environmental conditions noted in the ground truth data. For instance, BirdNET achieved 87.5% detection for howls under rainy conditions and with red deer (88.9%) in the background. Even for howls labeled as ‘very unclear’, BirdNET detected 50% (11/22). This strong recall, particularly in diverse noisy contexts, reinforces its utility for capturing a broad range of vocalizations.

In contrast Cry-Wolf generally exhibited lower overall recall, detecting 155 out of 260 howls (59.6% recall). Like BirdNET, Cry-Wolf also presented notably low precision across all datasets (0.005 overall), indicating a substantial generation of false positives. The model’s performance varied significantly with acoustic conditions; for instance, Cry-Wolf entirely missed all 8 howls in Dataset 3 (0% recall for that subset) and detected only 40% of faint howls.

BioLingual exhibited a slightly higher overall recall than Cry-Wolf, detecting 160 out of 260 howls (61.5% recall). However, like Cry-Wolf and BirdNET, BioLingual also presented consistently low precision across all datasets (0.005 overall), leading to a high number of false positives requiring human review. Its performance was also sensitive to environmental interferences, particularly performing poorly on howls occurring during rain (25% detection) and with bird background sounds. Despite these limitations, BioLingual demonstrated unique strengths in specific categories. Notably, it matched BirdNET’s high recall in Dataset 4 (89% for both models), and in the detection of very faint howls (50% for both models) and faint howls in windy conditions (83.3% for both models). This indicates BioLingual’s potential for distinguishing the characteristics of howls even in faint recordings.

When all three detection methods were combined—and a positive detection from at least one method was considered—the cumulative detection rate improved significantly. From the 260 manually annotated howls, 250 were detected by at least one of the AI models. The higher combined detection rate (96.2%) strongly underscores the power of a multi-model approach in maximizing true positive identification.

However, high recall comes at the cost of precision. All three models exhibited low precision (0.005–0.007), resulting in a substantial number of false positives. Crucially, this trade-off must be interpreted within the practical context of rare species monitoring, where missing a wolf (false negative) is far more costly than verifying a false detection. When viewed as a data reduction tool, the workload remains manageable: for BirdNET, the low threshold of 0.002 generated approximately 20,000 false detections, but this filters the 826.5 h of raw audio down to 24 h of review material. Similarly, BioLingual’s threshold of 0.94 reduced the review load to 84 h. This demonstrates that despite low precision, the system successfully reduces the listening task into a feasible amount.

A key finding is that there are no inherent trade-offs or disadvantages when running multiple AI models on the same dataset; instead, this strategy allows researchers to leverage the complementary strengths of each tool, optimizing for different research priorities (e.g., maximizing detection vs. minimizing manual review).

### 4.2. Broader Implications for Wolf Monitoring

AI-driven acoustic monitoring offers a powerful complement to traditional wolf monitoring methods, including DNA analysis from scat, camera trapping, and public observations [4].

The growing wolf population in Denmark [1,2] has made traditional intensive monitoring methods costly and largely reduced by 2025 [5,6,8]. Consequently, the national strategy now estimates wolf numbers based on pack counts, using a fixed conversion factor (e.g., 7 individuals per pack). This approach, however, introduces significant uncertainty due to variable pack sizes and challenges in identifying all active packs. Given these limitations and budget constraints that restrict intensive methods like DNA analysis to approximately 100 samples annually [7], AI-driven acoustic monitoring offers a promising, non-invasive, and cost-effective supporting tool for wolf population assessment [6,7]. By integrating acoustic data with other monitoring streams like GPS telemetry or scat collection, it can optimize resource allocation by prioritizing areas for more intensive methods, and, in time, may also enable the identification of individual wolves and puppies. AI-driven acoustic monitoring can significantly support this evolving national strategy across several key areas:

Enhanced Presence/Absence Detection and Territoriality: AI-driven acoustic surveys can provide continuous monitoring over large areas. This is especially useful for detecting wolf presence in new or suspected territories, thereby triggering targeted follow-up with DNA collection or camera traps. This aligns with the need for efficient data collection given reduced DNA sampling budgets. It can also indicate continued occupancy in known territories with less reliance on frequent genetic re-identification and contribute data to map activity hotspots within territories.

Supporting Pack-Based Estimation: While AI howl detection, even with strong recall, cannot yet directly count individuals or packs size, it can help confirm the presence of multiple vocalizing animals, suggestive of pack activity, thus supporting the identification of areas likely to contain packs. The detection of vocalizations labeled ‘pup’ howls by all models in our dataset, while limited, suggests the potential for AI to detect pup vocalizations, identifiable by distinct acoustic characteristics such as higher frequency energy [9] and shorter signals than adults [21]. Subject to expert verification, these detections could provide evidence of breeding activity, a key element in defining a “pack” for national estimates.

Efficiency and Resource Allocation: Automated detection drastically reduces the human effort required to examine vast amounts of audio data. While manual validation of AI-flagged events is still necessary given the overall low precision (F1-scores ranging from 0.01 to 0.014), the ability to focus review only on flagged clips is far more efficient than manual listening to all recordings.

Non-Invasive Data Collection: Acoustic monitoring is entirely non-invasive, avoiding any disturbance to the animals, which is a significant advantage for sensitive species. Additionally, wolves may be monitored without entering private grounds.

### 4.3. Future Development Opportunities

Current AI systems, while effective for howl detection, have limitations that present significant opportunities for future improvement and expanded application:

Improving Precision: A critical area for future AI development is the significant improvement of precision to reduce false positives. The very low precision values observed in this study (overall 0.007 for BirdNET, 0.005 for BioLingual and Cry-Wolf) highlight that despite good recall, the models generate a vast number of false positives. While the ‘cost’ (human screening time)of reviewing a single false positive is minimal, the sheer volume can still be substantial, limiting scalability, especially in acoustically diverse wild environments [19,22]. Reducing this false positive burden will make these tools even more efficient and widely adoptable for large-scale monitoring efforts [23].

Full Analysis of Additional Datasets: A crucial next step for this research involves conducting a full manual annotation and comprehensive comparative AI analysis on more datasets. This will confirm the preliminary observations from and allow for the generalization of our findings across varied Danish wolf habitats, providing a more robust understanding of model performance in different environmental contexts.

Individual Identification: While individual wolf identification was beyond the scope of this study, substantial research evidence indicates this represents a promising possibility for future AI development. The ‘from the same wolf ground truth note, while only occurring once, was detected by BirdNET, BioLingual and BirdNET, indicating that distinguishing individual vocalizations is possible. Larsen et al. (2022) [14] established individual wolf identification through acoustic analysis, achieving high classification accuracy using lone howls. Supporting research demonstrates that wolf packs maintain distinctive, stable vocal signatures over time [24,25], and individual wolves possess unique vocal characteristics suitable for identification [26]. Future AI systems could incorporate sophisticated “acoustic fingerprinting” techniques, analyzing complex acoustic parameters for individual recognition.

Howl Type Differentiation: The methodology can be further developed to distinguish different howl types, such as howls, growls, barks, whines, and whimpers [27]. Our detection of a ‘pup’ howl across methods suggests this capability. Young wolves vocalize less frequently and produce shorter signals than adults [21], and their howls concentrate acoustic energy at higher frequencies [10]. This differentiation is crucial for reproductive monitoring [10,28] and assessing reproductive success [21]. BioLingual, with its language model foundation, shows promise for this nuanced differentiation of howl types.

Environmental Context and Human Impact: The data included howls influenced by various environmental sounds such as rain, very noisy, very windy and airplanes. While BirdNET maintained high recall in many of these conditions, the performance of other models varied significantly, indicating the impact of environmental factors. AI models could be further developed to analyze whether animals reduce vocalization after human noise events, linking to human disturbance and nocturnality patterns [29]. Environmental factors, including landscape, topography, vegetation cover, and weather, significantly affect sound wave propagation, influencing detected frequencies and localization accuracy [9,14,25,26]. Future models could integrate these environmental variables to improve detection and interpretation, as wolves may modulate their vocalizations based on environmental conditions [26].

Optimal Monitoring Periods: Understanding the ecological and behavioral context of wolf howling is essential for efficient recording schedules. Wolf howls primarily serve territory defense, intra-pack contact, and social bonding purposes [10,13,25,30,31]. Howling activity exhibits clear seasonal and circadian patterns, with peak intensity during July through October, coinciding with pup rearing [5,13,14,27,32,33], and predominantly at night, especially from dusk to early morning [29,31]. These insights inform optimal recording schedules for maximizing detection efficiency and minimizing the number of false detections.

Applicability to Other Species: The underlying AI methods, particularly BirdNET, are versatile and applicable to numerous other animal species for efficient and accurate identification across vast audio datasets [22,23], demonstrating the broader utility of this technology beyond wolf monitoring.

With recall rates of 59.6% to 78.5% for individual AI systems, and a combined detection rate of over 96%, their cost-effectiveness and efficiency gains through reduced manual review time make them invaluable tools. This highlights that a cost-effective system, even without perfect accuracy, can provide substantial benefits for large-scale monitoring efforts.

## 5. Conclusions

This study evaluated the effectiveness of monitoring wolves by their howls using three AI-based methods—BirdNET, BioLingual, and Cry-Wolf—for detecting wolf howls in passive acoustic recordings from Klelund Dyrehave, Southern Jutland, Denmark. BirdNET achieved the highest recall of 78.5%, followed by 61.5% for BioLingual and 59.6% for Cry-Wolf, and a combined approach of the AI methods improved overall detection to a recall of 96.2%. The results demonstrate that while none of the models are currently suitable as standalone detectors due to their high false positive rates, they show significant potential as complementary human-aided data reduction tools.

While acoustic monitoring should not currently replace established methods such as DNA analysis or camera trapping, large-scale acoustic monitoring with AI filtering could function as a supplementary method for wolf monitoring in the future.

## Figures and Tables

**Table 1:** Glossary of Performance Metrics.

Metric	Definition
Precision	Proportion of positive detections that are actual wolf howls (TP/(TP + FP))
Recall	Proportion of actual wolf howls correctly detected by AI method (TP/(TP + FN)).
F1-score	Harmonic mean of precision and recall (2 × (Precision × Recall)/(Precision + Recall)).
Detections	Total number of instances a candidate wolf howl is identified, regardless of its accuracy (TP + FP).
Positive detections	Total number of individual audio segments containing a candidate wolf howl after meeting or exceeding the established confidence threshold.

**Table 2:** Comprehensive Performance Overview for BirdNET, Cry-Wolf, and BioLingual on Datasets.

Dataset	Metric	BirdNET	Cry-Wolf	Biolingual
1	Precision	6.6% (76/1154)	2.5% (67/2678)	0.8% (36/4409)
	Recall	74.5% (76/102)	65.7% (67/102)	35.3% (36/102)
	F1-Score	0.121	0.048	0.016
2	Precision	3.2% (33/1022)	0.7% (30/4569)	0.5% (31/5808)
	Recall	66% (33/50)	60% (30/50)	62% (31/50)
	F1-Score	0.062	0.013	0.011
3	Precision	0.4% (6/1341)	0 (0/3278)	0.2% (4/2113)
	Recall	75% (6/8)	0% (0/8)	50% (6/8)
	F1-Score	0.009	0	0.004
4	Precision	0.3% (89/25,460)	0.3% (58/19,798)	0.5% (89/17,924)
	Recall	89% (89/100)	58% (58/100)	89% (89/100)
	F1-Score	0.007	0.006	0.01

**Table 3:** Combined Performance Overview for BirdNET, Cry-Wolf, and BioLingual across Datasets.

Metric	BirdNET	Cry-Wolf	BioLingual
Precision	0.007 (204/28,977)	0.005 (160/30,254)	0.005 (155/30,323)
Recall	78.5% (204/260)	59.6% (155/260)	61.5% (160/260)
F1-Score	0.014	0.01	0.01

**Table 4:** AI Performance by Howl labels.

Note Type	Occurrences	BirdNET (Detected/Total)	Cry-Wolf (Detected/Total)	BioLingual (Detected/Total)
No label	32	28/32 (87.5%)	18/32 (56.3%)	15/32 (46.9%)
Rain	8	7/8 (87.5%)	6/8 (75.0%)	2/8 (25.0%)
Red deer	9	8/9 (88.9%)	7/9 (77.8%)	7/9 (77.8%)
Unclear	5	4/5 (80.0%)	2/5 (40.0%)	3/5 (60.0%)
Total	54	47/54 (87.0%)	33/54 (61.1%)	27/54 (50.0%)

**Table 5:** Performance pooled across the four datasets, evaluated with a 10 s symmetric alignment window.

Model	Recall (%)	Precision	False Positives	Strengths
BirdNET	78.5	0.007	28,773	87.5% during rain; 88.9% with deer rutting calls; 80% on unclear howls
Cry-Wolf	59.6	0.005	30,168	75% during rain; 77.8% with red deer sounds
BioLingual	61.5	0.005	30,094	Matched BirdNET’s 89% recall on Dataset 4; 77.8% with red deer; 60% on unclear howls; detected 50% where Cry-Wolf had 0%

## Data Availability

Data are available from the first author on request.

## Supplementary Materials

The following supporting information can be downloaded at: https://www.mdpi.com/article/10.3390/ani16020175/s1, Table S1: Ground truth howls with labels and percentage detected for BirdNET, BioLingual and CryWolf; Table S2: Glossary.

## Abbreviations

The following abbreviations are used in this manuscript:

AI	Artificial Intelligence
AZCF	Aalborg Zoo Conservation Foundation
SM4	Song Meter SM4 (Bioacustic recorder)
EU	European Union
FFT	Fast Fourier Transform
CNN	Convolutional Neural Network
FN	False Negative
FP	False Positive
Ff	Fundamental frequency
PAM	Passive Acoustic Monitoring
TN	True Negative
TP	True Positive
