# Supplementary material for: Bioacoustic Detection of Wolves Using AI (BirdNET, Cry-Wolf and BioLingual)
Source: Animals (Basel). 2026 Jan 7;16(2):175. doi: 10.3390/ani16020175 (PMC12838021; doi:10.3390/ani16020175)
Supplement: Supplementary file 1 [file animals-16-00175-s001.zip › animals-4074728-supplementary.pdf]

Supplementary

Table S1. Ground truth howls with labels and percentage detected for BirdNET, BioLingual and CryWolf.

Note Type	Occurrences	BirdNet (TRUE / Total)	BioLingual (TRUE / Total)	CryWolf (TRUE / Total)
(No Note)	74	68 (91.9%)	49 (66.2%)	68 (91.9%)
Other sound + rain	2	2 (100%)	0 (0%)	2 (100%)
Other sound at the end	1	1 (100%)	0 (0%)	1 (100%)
Other sound two times	1	1 (100%)	0 (0%)	1 (100%)
Another animal at the end	1	1 (100%)	0 (0%)	0 (0%)
bird	2	0 (0%)	0 (0%)	0 (0%)
birds	5	5 (100%)	0 (0%)	5 (100%)
From the same wolf	1	1 (100%)	1 (100%)	0 (0%)
Bark from dog	1	1 (100%)	1 (100%)	1 (100%)
Hoarse	1	1 (100%)	1 (100%)	1 (100%)
Puppy	1	1 (100%)	1 (100%)	1 (100%)
Red deer at the end	1	1 (100%)	1 (100%)	0 (0%)
Red deer	5	5 (100%)	3 (60.0%)	5 (100%)
With bird	2	2 (100%)	0 (0%)	0 (0%)
With red deer	1	1 (100%)	0 (0%)	1 (100%)
With duck	2	2 (100%)	1 (50.0%)	2 (100%)
Very noisy	3	1 (33.3%)	0 (0%)	0 (0%)
Very faint/unclear	22	11 (50.0%)	11 (50.0%)	6 (27.3%)
Very windy	1	1 (100%)	1 (100%)	0 (0%)
Maybe other sound at the same time	1	1 (100%)	1 (100%)	1 (100%)
Maybe other sounds at the same time	1	1 (100%)	1 (100%)	1 (100%)
Rain	20	20 (100%)	7 (35.0%)	18 (90.0%)
Rain + other sound at the end	1	1 (100%)	0 (0%)	1 (100%)
Rain + red deer	1	1 (100%)	1 (100%)	1 (100%)
Rain + red deer	1	1 (100%)	0 (0%)	1 (100%)
Strong/clear howl	10	10 (100%)	5 (50.0%)	10 (100%)
Two wolves	1	1 (100%)	1 (100%)	1 (100%)
Two wolves?	1	1 (100%)	1 (100%)	1 (100%)
Wolf?	1	1 (100%)	1 (100%)	0 (0%)
Faint/unclear	39	33 (84.6%)	24 (61.5%)	25 (64.1%)
Unclear + airplane	1	0 (0%)	0 (0%)	1 (100%)
Faint/unclear + bark from dog	1	1 (100%)	1 (100%)	1 (100%)
Faint/unclear + red deer	1	1 (100%)	1 (100%)	1 (100%)
Faint/unclear + red deer	6	5 (83.3%)	5 (83.3%)	4 (66.7%)
Red deer in the beginning	1	1 (100%)	1 (100%)	1 (100%)
Total Rows	**260**	**204 (78.5%)**	**160 (61.5%)**	**155 (59.6%)**

Table S2. Glossary

**Acoustic fingerprinting**	A technique for identifying unique acoustic characteristics or patterns in vocalizations that can distinguish between individual animals, similar to how human fingerprints are unique identifiers.
**Amplitude resolution**	The precision with which the magnitude of sound waves is digitally represented, measured in bits (e.g., 16- bit resolution provides 65,536 possible amplitude levels).
**Audio embeddings**	Mathematical vector representations of audio segments that capture acoustic features in a format suitable for machine learning analysis and comparison.
**Bioacoustics**	The scientific study of sound production, transmission, and reception in animals, including the ecological and behavioral contexts of animal vocalizations.
**Buffer window**	A time interval (in seconds) added before and after detected events to account for temporal discrepancies between automated detection and manual annotation boundaries.
**CNN (Convolutional Neural Network)**	A type of deep learning architecture particularly effective for analyzing data with spatial or temporal patterns, commonly used in image and audio processing.
**Confidence threshold**	A numerical cutoff point used by AI models to determine whether a detection is positive; detections with confidence scores above this threshold are classified as positive identifications.
**Cosine similarity**	A mathematical measure of similarity between two vectors, calculated as the cosine of the angle between them, commonly used to compare audio embeddings in AI models.
**Deep learning**	A subset of machine learning using neural networks with multiple layers to learn complex patterns and representations from data.
**F1- score**	The harmonic mean of precision and recall, providing a single metric that balances both measures of classifier performance (formula: 2 × (Precision × Recall) / (Precision + Recall)).
**False negative (FN)**	An actual wolf howl that was not detected by the AI system (missed detection).
**False positive (FP)**	A detection by the AI system that was not actually a wolf howl (incorrect detection).
**FFT (Fast Fourier Transform)**	A mathematical algorithm that converts time-domain audio signals into frequency- domain representations, enabling analysis of the spectral content of sounds.
**Fundamental frequency**	The lowest frequency component of a periodic sound wave, which largely determines the perceived pitch of the sound
**GPS telemetry**	A wildlife monitoring technique using satellite- based Global Positioning System technology to track animal movements and locations in real-time.
**Ground truth**	The definitive, manually verified dataset against which automated detection systems are evaluated; represents the actual correct answers for comparison.
**Language-audio model**	An AI system that can process and understand relationships between textual descriptions and audio content, enabling cross-modal analysis.
**Multivariate analysis**	Statistical techniques that analyze multiple variables simultaneously to identify patterns, relationships, or classifications within complex datasets.
**Passive acoustic monitoring**	A non-invasive wildlife survey method using automated recording devices to capture animal vocalizations without human presence or intervention.
**Precision**	The proportion of positive detections that are actually correct (formula: True Positives / (True Positives + False Positives)).

**Recall**	The proportion of actual positive cases that were correctly detected (formula: True Positives / (True Positives + False Negatives)); also known as sensitivity.
**Sampling rate**	The frequency at which analog audio signals are converted to digital format, measured in Hz or kHz (e.g., 44.1 kHz means 44,100 samples per second).
**Sonogram**	A visual representation of sound showing frequency content over time, with frequency on the vertical axis, time on the horizontal axis, and intensity represented by color or darkness.
**Spectral content**	The distribution of energy across different frequencies within a sound signal, revealing the acoustic characteristics of vocalizations.
**Transformer-based model**	A neural network architecture that uses attention mechanisms to process sequential data, originally developed for natural language processing but adapted for audio analysis.
**True negative (TN)**	A correctly identified absence of a wolf howl (correct rejection).
**True positive (TP)**	A correctly identified wolf howl (correct detection).
**Zero shot classification**	An AI capability that allows models to classify or identify categories they were not explicitly trained on, using learned representations to generalize to new classes.
